# CROP: a CRISPR/Cas9 guide selection program based on mapping guide variants

**DOI:** 10.1038/s41598-021-81297-2

**Published:** 2021-01-15

**Authors:** Victor Aprilyanto, Redi Aditama, Zulfikar Achmad Tanjung, Condro Utomo, Tony Liwang

**Affiliations:** Plant Production and Biotechnology Division, Department of Biotechnology, PT SMART Tbk, Jl. Raya Cijayanti Kp. Pasir Maung RT 004 / RW 006; Babakan Madang, Bogor, 16810 Indonesia

**Keywords:** Bioinformatics, Biotechnology

## Abstract

The off-target effect, in which DNA cleavage was conducted outside the targeted region, is a major problem which limits the applications of CRISPR/Cas9 genome editing system. CRISPR Off-target Predictor (CROP) is standalone program developed to address this problem by predicting off-target propensity of guide RNAs and thereby allowing the user to select the optimum guides. The approach used by CROP involves generating substitution, deletion and insertion combinations which are then mapped into the reference genome. Based on these mapped variants, scoring and alignment are conducted and then reported as a table comprising the off-target propensity of all guide RNAs from a given gene sequence. The Python script for this program is freely available from: https://github.com/vaprilyanto/crop.

## Introduction

Clustered regularly interspaced short palindromic repeats (CRISPR) is a versatile genome editing tool which relies on the activity of CRISPR-associated (Cas) enzymes to break the DNA strand guided by short 20-nucleotide RNA sequence also known as a guide^1–4^. This RNA-guided nuclease (RGN) system has been widely used to knockout or edit genomic locations across various organisms, notably human, animals, and crop plants. However, this system has one caveat in which it gives off-target cleavage, resulting double-strand break (DSB) at locations other than targeted ones^5,6^.

A number of efforts has been conducted to minimalize this off-target effects, ranging from designing high fidelity Cas9^7–9^ to selecting high specificity guide RNAs (gRNA)^2,10,11^. In the latter approach, the specificity of 20-nucleotide gRNA could be predicted based from the base types and positions in the guide sequence^2^. This observation leads to a number of off-target prediction webserver programs, such as CCTop^12^, Cas-OFFinder^13^, CRISPOR^14^, CRISPR-PLANT^15^, CRISPR-P^16^ and others which are widely used to select gRNA with high specificity. However, the availability of the reference genomes in those webservers often restricts one to predict the off-target of a given guide at organism which genome is unavailable. In this study, we introduced CRISPR Off-target Predictor (CROP), a program for guide RNA off-target prediction which allows the user to use own’s genome of interest. The approach used in CROP is by creating guide variants via substitution, deletion or insertion schemes up to four positions along the twenty nucleotide-long gRNA sequence. This approach is designed to simulate DNA:RNA heteroduplex pairing with mismatch, RNA and DNA bulges. The resulting variants from each gRNA will subsequently be mapped to the reference genome, in which the mapped variants are then used to estimate the off-target propensity score of the corresponding gRNA.

## Results

### Total combination of a guide’s unique variants

In order to list all the possible off-targets bearing few substitutions, deletions or insertions for one guide, CROP produces all possible positional combinations of a guide. Under the substitution scheme the positional combination took place across the whole guide’s length, where the bases at these positions were substituted into another bases, creating a total of unique substitution variants ${S}_{U}$ according to (1):

1 $${S}_{U}=\sum_{i=1}^{r}{3}^{i}\left(\genfrac{}{}{0pt}{}{n}{i}\right)$$

where $1\le r\le n$ is the number of substituted position and $n$ is the number of nucleotides subjected for substitution. For a 20-nucleotide-long guide sequence, this substitution scheme will create 424,996 unique variants. Since this scheme generates variants with sequence length equal to the original guide, the sets of variants are nested, with the set generated through lower $r$ value is a subset of higher $r$ value. This observation was then used to modify the corresponding script by only generating variants from the highest $r$ value, which was 4.

In the deletion scheme the deletion was carried out towards 19 out of 20 nucleotides along the guide’s sequence, avoiding 5′-end nucleotide deletion which will create 19-nucleotide subsequence identical to the original guide. The total unique variants generated from this deletion scheme (${D}_{U}$) can be calculated according to (2):

2 $${D}_{U}=\sum_{i=1}^{r}\left(\genfrac{}{}{0pt}{}{n-1}{i}\right)$$

where $1\le r\le n$ is the number of substituted position and $\left(n-1\right)$ is the number of nucleotides subjected for deletion. However, the above formula only provides the maximum number of unique deletion variants which could be generated without specifying the exact number. Using the above formula, at most 5035 unique variants could be generated from a 20-nucleotide guide sequence. The exact number of unique variants was difficult to calculate since different sequences yields different unique deletion variants. It is thought that the occurrence of $r$-nucleotide repeats affect this number. As for example, ${D}_{U}$ for a guide comprising solely of A’s is 4, while guide AAAAATTTTTGGGGGCCCCC and ATGCGTCATCAGACGTAGTC would give ${D}_{U}$ 69 and 4647, respectively.

Then in the insertion scheme, a combination of four nucleotides were inserted in positions between the two nucleotides in guide sequence plus one position between the guide’s 3′-end and the first PAM site, giving a total of 20 sites available for insertion. The total unique variants generated from this scheme can be calculated according to (3):

3 $${I}_{U}=r+\sum_{i=1}^{r}\sum_{j=1}^{i}{3}^{j}\left(\genfrac{}{}{0pt}{}{n-1+i}{j}\right)$$

where $1\le r\le n$ is the number of substituted position and $\left(n-1+i\right)$ is the number of sites subjected for insertion. In opposite to the deletion scheme, the variants generated in this insertion scheme had longer sequences. The total unique combination under this scheme was also larger than substitution and deletion schemes as was calculated by (3). The total unique insertion variants generated from one 20-nucleotide guide was 813,160, comprising of 61; 1,954; 43,726 and 767,419 for 1, 2, 3 and 4 insertions, respectively.

In total, these three schemes would give 1,243,190 unique variants from one guide. In addition to NGG PAM, CROP also considers NAG PAM which is reported to yield Cas9 cleavage albeit with lower probability^2,10^. Therefore, with a combination of NRG PAM (R = A or G), the total unique variants would increase eight folds to 9,945,520.

## CROP runtime and output delivery

**Figure 1:**
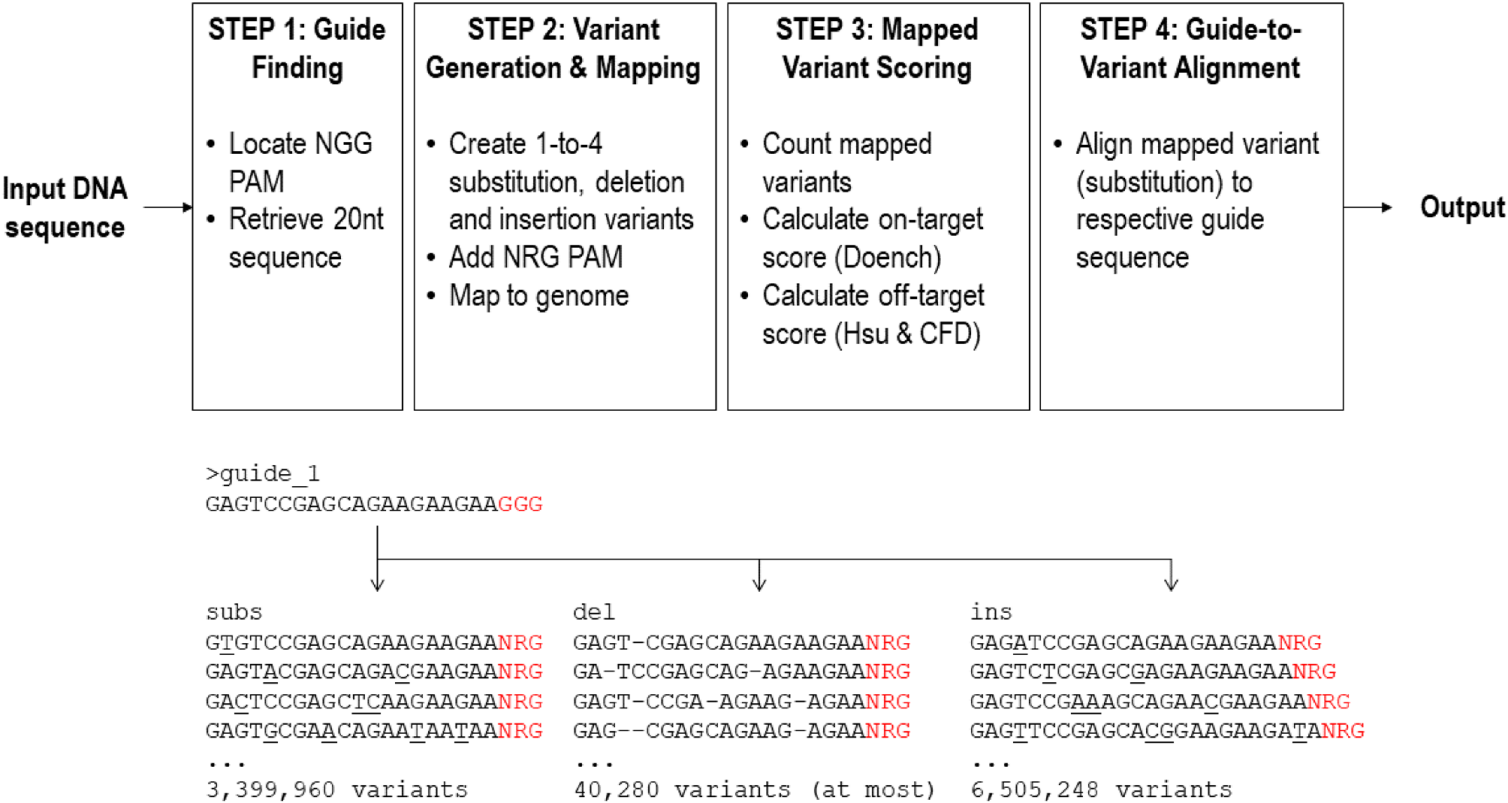
CROP working scheme (top) and generation of variants from a 20-nucleotide guide sequence (bottom).

Using a standard desktop computer (i5, 8 Gb RAM) CROP required more or less 100 s from guide finding until guide-to-variant alignment steps (Fig. 1). A typical gene fragment giving 100 guide sequences would require around 3 h runtime. Although the runtime could be improved using more powerful cores, it was still considered less efficient compared to other web-based CRISPR programs, which usually produces results in minutes. The main limiting factor for CROP runtime was likely in the variant generation step which requires the program to generate almost ten million variants per guide. Several approaches could be attempted to improve CROP’s efficiency, including the usage of higher number of computer cores or considering subsets among the total variants which are frequently observed to cause off-targeting in Cas9.

Using more computer cores could increase the number of parallel processes executed at one time. A standard desktop computer (i5) contains four cores which means that it could predict four guides at one time. A standard workstation with 88 cores could predict 22 times more guides at one time compared to the standard desktop. On variant subset consideration, several studies on CRISPR applications^1,2,17^ reported that the actual off-targets are locations in which the sequence contain less or no mismatches at the PAM proximal region (ten nuelcotides adjacent to PAM). This region is therefore an important determinant for Cas9 activity. Assuming that a Cas9-gRNA complex would only cleave locations given a perfect match in the PAM proximal, then the variant set could be restricted to only containing sequences with mismatches at PAM distal region (nucleotides 11–20 adjacent to PAM). With such restrictions, the time needed to create guide variants and overall prediction process will be faster.

For each queried DNA sequence, CROP give a result in four directories, namely: (i) align, containing alignment between the guide sequence with its mapped variants; (ii) ot_maps, containing sam-formatted file listing mapped guide variants; (iii) single_guides, containing single-fasta files of single-guide RNA which could be used for mFold RNA loop prediction^18^; and (iv) outfiles, containing score table, raw sequence, and multi-fasta files. The guides in raw sequence file are written by including additional bases in N_4_-guide-NGG-N_3_ format as input for Azimuth score calculation in https://crispr.ml/ website^11,19^. The score file contained a number of information for each guide, including GC content, Doench on-target score, map counts, Hsu & CFD off-target score, and number of on target count. All on- and off-target scores are set to a 0–100 scale. A log file was also written in the result directory, containing all the activities that CROP carried out towards the corresponding DNA sequence.

### Highly variable on- and off-target scores among PATE Exon-1 guides

**Figure 2:**
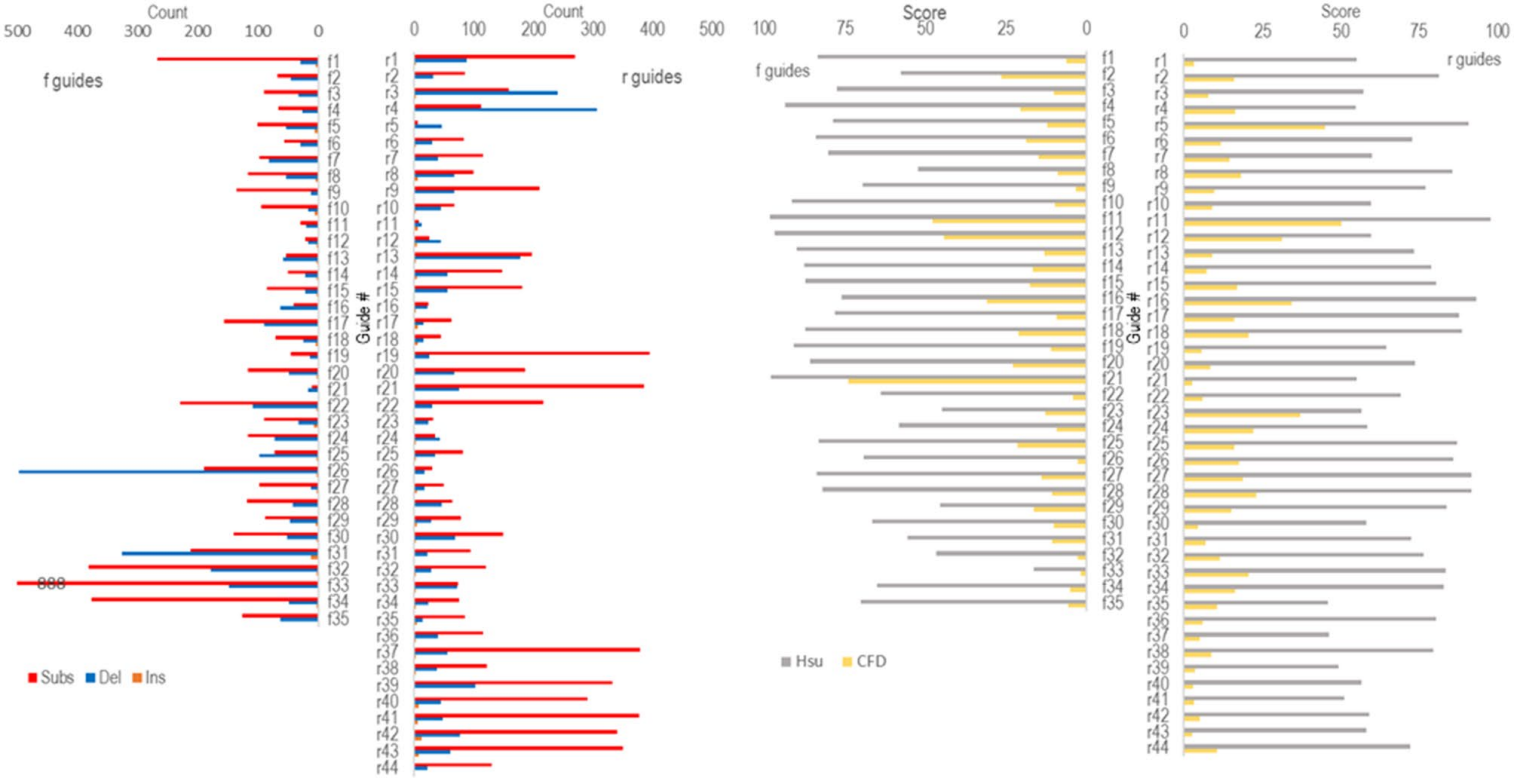
CROP prediction on guides derived from oil palm PATE exon-1 gene (XM_010926998). Left: off-target counts of mapped substitution, deletion and insertion variants; right: off-target Hsu and CFD scores derived from mapped substitution variants. Higher score translates to lower off-target propensity.

Among nearly 10 million variants per guide, only a tiny fraction of which mapped to the oil palm EG5 reference genome. For PATE exon-1 gene (XM_010926998), these fractions ranged from 0.0003 to 0.01% which were dominated by substitution and deletion variants. CROP produced 79 guides from PATE exon-1 gene, comprising 35 forward and 44 reverse guides (Fig. 2; Supplementary Table S1). Based on these counts, the mapped insertion variants (Ins) had the lowest counts among all three. Both mapped substitution (Subs) and deletion (Dels) variants dominated the counts with majority of the guides had higher counts on the former. These mapped counts varied widely from as low as zero in mapped insertion variants to near 900 in mapped substitution variants. Similar to off-target counts, the Hsu and CFD off-target scores also displayed wide variations among the guides with the former generally being larger than the latter. Such score difference might be related with the employment of different off-target databases by both scoring system. The database used in CFD calculation was inferred from a larger dataset compared to the Hsu’s^2,11^. This might hint that CFD is a better predictor in regards of off-target propensity of guide RNA^20^.

Ideally, guide sequences to be used in genome editing applications should be ones which exhibit high on-target while maintaining low off-target capabilities. However, as shown in the case of PATE exon-1, an opposite relationship was observed between on-target (Doench^11^) and off-target (Hsu^2^ and CFD^11^) scores. Guides possessing high off-target scores, those that possess high specificity, tend to give poor on-target scores and vice versa. Such opposite relationship tends to limit the search for optimal guides and therefore a compromise has to be made. For example, guide r5 (Fig. 2; Supplementary Table S1) was considered to be the best among all 79 guides since it possess a high on-target and adequate off-target scores. Other guide such as guide f21 also possessed high off-target scores but scored poorly on on-target score. To avoid inability of Cas9 cleavage cause by such guide, it is recommended for all the selected guides to be in vivo tested on a cell or protoplast culture systems. By this way, the actual on- and off-target can be assessed more thoroughly through next generation sequencing technology.

### Different off-target numbers between guide-seq and CROP

GUIDE-Seq has been regarded as the gold standard in measuring off-target propensity of guide RNAs. In order to compare CROP prediction with GUIDE-Seq, we predicted the off-target propensity of ten guide RNAs previously used in the GUIDE-Seq study and compared the results. CROP predicted greater numbers of off-target locations compared to what had been shown by GUIDE-Seq for each guide RNA (Supplementary Table S2). The CROP-to-GUIDE-Seq number ratio ranges from 6 to 293, indicating that CROP gave off-target locations far higher than GUIDE-Seq results.

## Discussion

Predicting off-target propensity of a guide RNA (gRNA) in CRISPR/Cas9 applications is an important step in addition to making sure its on-target capability. Methods designed to measure off-target potential of a gRNA in vivo (GUIDE-seq^17^) and in vitro (CIRCLE-seq^21^) have both shown that there are many sites which serve as potential off-target in the genome. CROP adds to a collection of publicly available CRISPR/Cas9 off-target prediction software. It differs to the other software based on two factors: (1) the number of guide variants mapped into the target genome and (2) the flexibility of CROP to incorporate any genome as references.

In terms of guide variant number, most off-target prediction programs measure the off-target propensity by mapping gRNA against the genome while allowing several site differences between the two. Unfortunately, such mapping process could not be executed thoroughly by the mapping software since there is a limitation regarding the maximum number of reported genomic sites where the gRNA could map. This behavior tends to underestimate the number of potential off-target sites in the genome as the Cas9 enzyme could tolerate up to four base mismatches within 20 base gRNA-DNA pair. CROP overcomes this issue by creating one up to four substitutions, deletions and insertions at every possible site combination along the 20-bp gRNA sequence. This produces nearly ten million sequence variants from a single gRNA which are later mapped against the reference genome. The mapping itself does not allow any mismatches between each gRNA variant to the genome considering such mismatches have already been simulated in the production of gRNA variants. This approach ensures a thorough mapping result of a guide which represents its actual off-target propensity.

Most off-target prediction program provides their services with a limited number of reference genomes. The commonly cited guide RNA off-target prediction programs such as CRISPOR^20^, CHOPCHOP^22^ and CRISPR-P^23^ currently have only 610, 162 and 75 genomes to select. Although these numbers are growing, one still might not get the genome of interest. At the time of this study, we could not find oil palm (*Elaeis guineensis* Jacq.) genome in these webservers and therefore could not predict the off-target propensity of guide RNAs targeted for oil palm genome. This poses a difficulty when a prediction would be made without any available reference target genomes. As stand-alone software, CROP provides flexibility by allowing the users to use their own reference genome to run the off-target prediction. CROP only requires the user to index the fasta-formatted genome sequence into bowtie (.bwt) format and then place the indexed files in the genome folder provided in the software. In order to speed up its process we amended CROP with parallel processing of multiple input sequences. This parallelization process makes CROP longest runtime of one execution depends only towards one input sequence with the most numbers of gRNAs. The use of multiple-core computer such as workstation is therefore recommended to make a faster off-target prediction of several input sequences.

A test case using first exon of oil palm PATE gene revealed that all guides must have one of its substitution and/or deletion variants mapped to other locus in the genome. In a test case using first exon of oil palm PATE gene, it is interesting to observe that the numbers of mapped insertion variants were significantly smaller than substitution and deletion variants (Fig. 2). This difference might be related to the smaller probability to find an exact match in the longer sequence of insertion variants as opposed to the higher matching probability in the shorter deletion variants. The higher mapped substitution variants compared to deletion and insertion in most guide RNAs might indicate that there is some degree of sequence similarity between the exonic sequence (like this PATE exon 1 gene) and other regions across the genome, but further investigation is still required. Additionally, we can also observe that all guides must have at least one of its substitution and/or deletion variants mapped to other locus in the genome. If this observation is translated as off-target potential there would be no gRNA ideal enough to satisfy the zero off-target requirement. However in real applications, the actual off-target tends to be lower than what has been predicted by off-target prediction software, including CROP. For example, a simple comparison between CROP prediction and GUIDE-seq result of ten gRNAs obtained from Tsai and colleagues^17^ revealed that the majority numbers of actual off-target sites were less than 10% of what had been predicted by CROP (Supplementary Table S2). This observation might be attributed by but not limited to inaccessible DNA region or low tolerance of the Cas9 ribonucleoprotein itself. More actual CRISPR/Cas9 off-target experiments are clearly necessary to better model CROP algorithm in the future.The fact that Hsu and CFD scorings were based on gene editing study of mouse and human cells^2,11^ might pose a question whether they could be applied in other organisms. Using such scoring systems for other organisms (e.g. oil palm in our case) might give discrepancies between which is predicted as a good guide RNA candidate and the actual good one, considering a distant relationships between oil palm to mouse and human. Further study might be focused to address the prediction of a minimum off-target guide RNA candidates using off-target data from the genome of target organism itself. Ideally, a benchmark of actual gRNA off-target measurement is advised to be taken in order to calibrate CROP’s accuracy. Running GUIDE-seq and CIRCLE-seq for in vivo and in vitro off-target measurement, respectively, would set a good benchmark for this. Additionally, a new more affordable technique for in vitro off-target measurement using Nanopore sequencing technology (Nano-OTS)^24^ could also be used to provide similar data for future experiments. In conclusion, CROP can be readily downloaded from GitHub server (https://github.com/vaprilyanto/crop) and used as stand-alone software in any currently available computers to provide gRNA off-target propensity. This might serve as a starting point for gRNA design prior to CRISPR/Cas9 genome editing applications. A test using oil palm PATE exon-1 gene revealed that some guides, such as f11, f12 and r11 display high Hsu and CFD scores among the others and thus they could be recommended.

## Methods

### Generating guide variants and mapping to genome

All the steps conducted in CROP are summarized in Fig. 1. For guide finding step, CROP lists all the available 20-nucleotide guide sequences from the input DNA sequence. Then in variant generation step, a number of variants were generated from each guide. This step was carried out through generating all possible positional combinations ranging from one up to four nucleotides across guide’s length. These positional combinations were then used in three subsequent schemes of guide modifications, namely substitution, deletion and insertion. In substitution scheme guide variants were created by replacing bases at positions given by the positional combinations into all possible combinations of DNA bases. In deletion scheme, the bases at these positions were deleted, giving shortened variants from the original guide. The last scheme uses these positional combinations to insert bases into the guide sequence to yield longer variants as the result. In addition to base modifications inside the 20-nucleotide guide sequence, eight combinations of NRG protospacer adjacent motif (PAM) consisting of AGG, TGG, GGG, CGG, AAG, TAG, GAG, and CAG were added as prerequisites for *Streptococcus pyogenes* Cas9 (SpCas9) cleavage, therefore multiplies the total variants to eight fold. All these variants were then mapped towards the reference genome using Bowtie read-mapping^25^ using zero mismatches (-v 0) and reporting all available mapped locations per variant (-a). The mapping-result was reported as a sequence alignment map (.sam)-formatted file which serves as the base for further scoring and alignment processes.

### Scoring functions, alignment and parallelization

To assess off-target propensity CROP uses counts mapped variants along with Hsu^2^ and CFD^11^ off-target scoring calculations. The mapped variant counts included mapped substitution, deletion and insertion variants. The python scripts for both scoring systems were adopted from CRISPOR program^14^ through GitHub (https://github.com/maximilianh/crisporWebsite) with minor modifications including code simplifications and CFD score re-scaling. Using only mapped substitution count, Hsu and CFD off-target scores were then calculated for each guide-variant pair. The usage of only mapped substitution was due to the requirement of the same sequence length between guide and variants. After that, cumulative Hsu and CFD scores among all guide-variant pairs for one guide was calculated according to each aggregate scores^16,23^.

CROP also included on-target scores for each guide, including Doench score^10^ as well as 30-nucleotide sequence bearing N_4_–N_20_–NGG–N_3_ (N_20_ = 20nt guide sequence) format for calculating Azimuth score through Machine learning-based end-to-end CRISPR/Cas9 guide design (https://crispr.ml/) website^11,19^. In addition to scoring, the program also aligned all the mapped variants to the original guide sequence and marked mismatches, highlighting the mismatches for all pairs. In order to automate all the process, a linux script (core.sh) was written to develop a pipeline which consists of four steps, namely: (i) finding guide sequences, (ii) creating guide variants and mapping to reference genome, (iii) variants scoring and (iv) variant alignment. Another Linux script (crop.sh) was also written as the main command file to allow running multiple core.sh in parallel, thereby allowing the user to conduct multiple CRISPR guide analyses in a single operation.

### Test cases

In order to test its performance, CROP was tested for predicting optimum guides for targeting palmitoyl-acyl carrier protein thioesterase gene (PATE; GenBank accession number XM_010926998) in African oil palm (*Elaeis guineensis*). The oil palm genome (assembly EG5) was downloaded from NCBI Genome database and used as the reference genome for mapping. In the second test case, we tested CROP to predict the off-target propensity of ten guide RNAs previously used in GUIDE-Seq study^17^. All guide variants produced from these ten guides were mapped against human genome reference (GrCh37) and the numbers of off-target obtained from substitution guide variants were compared with GUIDE-Seq off-target data.

## Supplementary Information

Supplementary Information

## References

[1] Jinek M, Chylinski K, Fonfara I, Hauer M, Doudna JA (2012). A programmable dual-RNA-guided DNA endonuclease in adaptive bacterial immunity. Science (80-)..

[2] Hsu PD (2013). DNA targeting specificity of RNA-guided Cas9 nucleases. Nat. Biotechnol..

[3] Sternberg SH, Redding S, Jinek M, Greene EC, Doudna JA (2014). DNA interrogation by the CRISPR RNA-guided endonuclease Cas9. Nature.

[4] Barrangou R, Doudna JA (2016). Applications of CRISPR technologies in research and beyond. Nat. Biotechnol..

[5] Wu X, Kriz AJ, Sharp PA (2014). Target specificity of the CRISPR-Cas9 system. Quant. Biol..

[6] Tsai SQ, Joung JK (2016). Defining and improving the genome-wide specificities of CRISPR–Cas9 nucleases. Nat. Rev. Genet..

[7] Slaymaker IM (2016). Rationally engineered Cas9 nucleases with improved specificity. Science (80-)..

[8] Kleinstiver BP (2016). High-fidelity CRISPR-Cas9 nucleases with no detectable genome-wide off-target effects. Nature.

[9] Vakulskas CA (2018). A high-fidelity Cas9 mutant delivered as a ribonucleoprotein complex enables efficient gene editing in human hematopoietic stem and progenitor cells. Nat. Med..

[10] Doench JG (2014). Rational design of highly active sgRNAs for CRISPR-Cas9-mediated gene inactivation. Nat. Biotechnol..

[11] Doench JG (2016). Optimized sgRNA design to maximize activity and minimize off-target effects of CRISPR-Cas9. Nat. Biotechnol..

[12] Stemmer M, Thumberger T, Del Sol KM, Wittbrodt J, Mateo JL (2015). CCTop: An intuitive, flexible and reliable CRISPR/Cas9 target prediction tool. PLoS ONE.

[13] Bae S, Park J, Kim JS (2014). Cas-OFFinder: A fast and versatile algorithm that searches for potential off-target sites of Cas9 RNA-guided endonucleases. Bioinformatics.

[14] Haeussler M (2016). Evaluation of off-target and on-target scoring algorithms and integration into the guide RNA selection tool CRISPOR. Genome Biol..

[15] Minkenberg B, Zhang J, Xie K, Yang Y (2018). CRISPR-PLANT v2: An online resource for highly specific guide RNA spacers based on improved off-target analysis. Plant Biotechnol. J..

[16] Lei Y (2014). CRISPR-P: A web tool for synthetic single-guide RNA design of CRISPR-system in plants. Mol. Plant.

[17] Tsai SQ (2015). GUIDE-seq enables genome-wide profiling of off-target cleavage by CRISPR-Cas nucleases. Nat. Biotechnol..

[18] Zuker M (2003). Mfold web server for nucleic acid folding and hybridization prediction. Nucleic Acids Res..

[19] Listgarten J (2018). Prediction of off-target activities for the end-to-end design of CRISPR guide RNAs. Nat. Biomed. Eng..

[20] Concordet JP, Haeussler M (2018). CRISPOR: Intuitive guide selection for CRISPR/Cas9 genome editing experiments and screens. Nucleic Acids Res..

[21] Tsai SQ (2017). CIRCLE-seq: A highly sensitive in vitro screen for genome-wide CRISPR-Cas9 nuclease off-targets. Nat. Methods.

[22] Montague TG, Cruz JM, Gagnon JA, Church GM, Valen E (2014). CHOPCHOP: A CRISPR/Cas9 and TALEN web tool for genome editing. Nucleic Acids Res..

[23] Liu H (2017). CRISPR-P 2.0: An improved CRISPR-Cas9 tool for genome editing in plants. Mol. Plant.

[24] Höijer I (2020). Amplification-free long read sequencing reveals unforeseen CRISPR-Cas9 off-target activity. bioRxiv.

[25] Langmead B, Trapnell C, Pop M, Salzberg SL (2009). Ultrafast and memory-efficient alignment of short DNA sequences to the human genome. Genome Biol..

